# Graph distance for complex networks

**DOI:** 10.1038/srep34944

**Published:** 2016-10-11

**Authors:** Yutaka Shimada, Yoshito Hirata, Tohru Ikeguchi, Kazuyuki Aihara

**Affiliations:** 1 Faculty of Engineering, Tokyo University of Science, 6-3-1 Niijuku, Katsushika-ku, Tokyo 125-8585, Japan; 2 Institute of Industrial Science, the University of Tokyo, 4-6-1 Komaba, Meguro-ku, Tokyo 153-8505, Japan

## Abstract

Networks are widely used as a tool for describing diverse real complex systems and have been successfully applied to many fields. The distance between networks is one of the most fundamental concepts for properly classifying real networks, detecting temporal changes in network structures, and effectively predicting their temporal evolution. However, this distance has rarely been discussed in the theory of complex networks. Here, we propose a graph distance between networks based on a Laplacian matrix that reflects the structural and dynamical properties of networked dynamical systems. Our results indicate that the Laplacian-based graph distance effectively quantifies the structural difference between complex networks. We further show that our approach successfully elucidates the temporal properties underlying temporal networks observed in the context of face-to-face human interactions.

Describing real complex systems as coupled dynamical systems is a basic and powerful approach for enhancing our understanding of their dynamical properties. If we can appropriately evaluate the distance between graphs or networks, this distance can be used as a fundamental and effective tool to characterise differences in the dynamical properties of two different coupled dynamical systems, because couplings or network structures affect collective behaviour of the coupled dynamical systems including convergence of consensus dynamics^1^,^2^,^3^, controllability of coupled linear systems^4^,^5^,^6^,^7^,^8^, and basin stability of coupled, especially nonlinear, dynamical systems^9^,^10^.

Although several definitions of the distance between networks have been proposed^11^, they all simply focus on the differences in the number of nodes and links. For example, the Hamming distance is the sum of the simple differences between the adjacency matrices of two graphs^11^, and the graph edit distance is the minimum cost for transforming one graph into another by deletions and insertions of nodes and links^12^. These distances simply focus on the differences in the number of nodes and links, and lack the perspective of both dynamical and structural properties of complex networks. Here, we propose a graph distance for complex networks that reflects both their structural and dynamical properties, focusing on the Laplacian matrix that represents the coupling topology. We define a graph distance between networks through comparing their Laplacian matrices. Because the Laplacian matrix contains significant information about the structural and dynamical properties of networks, the Laplacian-based graph distance directly reflects the differences in both the structural and dynamical properties characterised by the Laplacian matrix. We show that the Laplacian-based graph distance is effectively applied to mathematical models and several real networks, even in a situation where their sizes differ from one another. We further apply the proposed distance to a real temporal network whose structure changes with time^13^,^14^. Our results indicate that the distance for complex networks is a key concept for understanding the dynamical properties of temporal networks.

## Graph Distance for Complex Networks

Let *A* be an *N* × *N* adjacency matrix of a given undirected network in which *A*_*ij*_ = 1 if the node *i* connects to the node *j* and *A*_*ij*_ = 0 otherwise. The Laplacian matrix associated with the adjacency matrix *A* is defined by


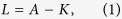


where *K* is the diagonal matrix, whose *i*th diagonal element is the degree of the *i*th node *k*_*i*_. The Laplacian matrix appears in several contexts of network science, such as detecting community structures in the networks^15^,^16^, consensus dynamics^1^, and describing diffusive coupling of nonlinear dynamical systems^9^,^10^. For example, a network consisting of connected *N* identical dynamical systems is governed by


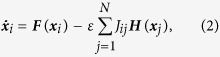


where ***x***_*i*_ is the multidimensional state vector of the *i*th dynamical system, *ε* is the coupling strength, ***F*** determines the intrinsic dynamics of each isolated uncoupled dynamical system, *J*_*ij*_ is a coupling coefficient which satisfies 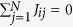, and ***H*** is the coupling function that defines the dynamics of the links. Equation (2) covers several coupling forms and indicates that the Laplacian matrix is crucial for estimating the stability of the various coupled dynamical systems^9^. In this paper, even though we mainly focus on the situation where the coupling matrix *J* = {*J*_*ij*_} is just the Laplacian matrix *L*, our approach can be naturally extended to other types of the coupling matrix *J*.

On the other hand, the detection problem of communities in networks is formulated as one of discrete optimisation problems. A well-known technique for detecting communities is spectral partitioning based on the direct relation between the structural properties of the network and the spectrum of the Laplacian matrix^16^. In this sense, the Laplacian matrix reflects both structural and dynamical properties of the networked dynamical systems.

**Figure 1:**
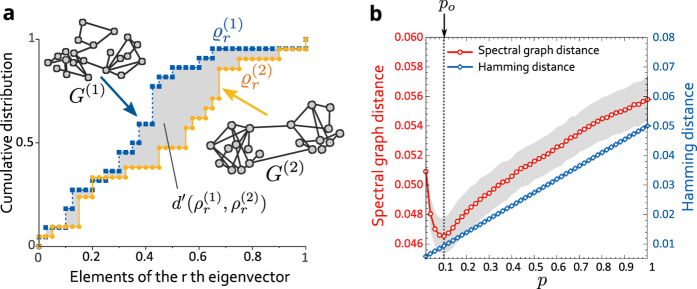
(**a**) Schematic diagram of the spectral graph distance described by Eqs (3) and (5). The distributions 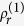 and 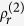 are obtained from the *r*th eigenvectors of the Laplacian matrix *L*^(1)^ corresponding to the network *G*^(1)^ and *L*^(2)^ corresponding to *G*^(2)^. (**b**) The spectral graph distances and the Hamming distances between the networks *G*(*p*_*o*_) and *G*(*p*) generated from the WS model, where the value of *p* is varied and *p*_*o*_ takes the fixed constant value (*p*_*o*_ = 0.1). The number of nodes *N* = 500, and each node has on average *k* = 10 links. The grey area indicates ± standard deviation.

In our approach utilising the spectrum of the Laplacian matrix, we propose a graph distance between two networks by calculating the distance between the spectra of the corresponding Laplacian matrices. Let *G*^(*i*)^ be the *i*th network with *N*^(*i*)^ nodes and *L*^(*i*)^ be its Laplacian matrix. The Laplacian matrix *L*^(*i*)^ is rewritten by 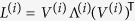, where Λ^(*i*)^ is the diagonal matrix 
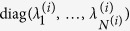
, 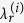 is the *r*th eigenvalue 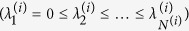, 
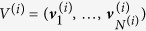
 and 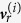 is the eigenvector corresponding to the *r*th eigenvalue of *G*^(*i*)^. The Laplacian matrix is a semi-definite matrix, whose smallest eigenvalue, *λ*_1_, is zero and the eigenvector corresponding to *λ*_1_ is **1** which is a column vector with *N* ones. Let 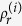 be the distribution of the elements in the *r*th eigenvector of the *i*th network. Given two networks *G*^(*i*)^ and *G*^(*j*)^, calculating the distance between pairs of the distributions 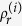 and 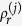, we define the distance between the two networks as


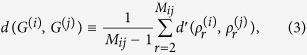


where 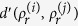 is the distance between 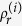 and 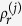, and 1 < *M*_*ij*_ ≤ min(*N*^(*i*)^, *N*^(*j*)^). Throughout this paper, *M*_*ij*_ = min(*N*^(*i*)^, *N*^(*j*)^). To calculate 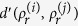, we introduce the Kruglov distance with the absolute value function from the viewpoint of computational convenience (see Methods and Supplementary Information). On the right-hand side of Eq. (3), 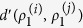 is not included in the summation because both eigenvectors corresponding to zero eigenvalues are 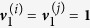, and do not give information concerning differences between two networks. Figure 1a shows the schematic diagram of Eq. (3). We call the distance defined by Eq. (3) the *spectral graph distance* (See Supplementary Information for detail of properties of the spectral graph distance).

## Results and Discussion

### Classifying network structures

To evaluate how the spectral graph distance performs, we first apply our method to the Watts–Strogatz (WS) model, which can generate small-world and random networks by rewiring edges in the initial ring lattices with a probability *p*^17^. If *p* = 0, the WS model generates ring lattices, but if *p* = 1, the WS model generates random networks. In the experiments, a network *G*(*p*_*o*_) is generated from the WS model with a fixed rewiring probability, *p*_*o*_, in advance. We then calculate distances between *G*(*p*_*o*_) and networks *G*(*p*), which are generated from the WS model with several values of the rewiring probability, *p*. If the proposed distance appropriately evaluates the distance between *G*(*p*_*o*_) and *G*(*p*), *d*(*G*(*p*_*o*_), *G*(*p*)) should take a minimal value when *p* = *p*_*o*_.

Figure 1b shows the results of the distances between *G*(*p*_*o*_) and *G*(*p*). Here, the spectral graph distance takes its minimum value when *p*_*o*_ ≈ *p*. On the other hand, the Hamming distance does not take its minimum value even when the value of *p* is close to that of *p*_*o*_. This result clearly shows that our distance effectively evaluates the difference between the two networks, whereas a simple distance focusing on the number of nodes and links cannot appropriately reflect the differences between the networks’ structural properties.

**Figure 2:**
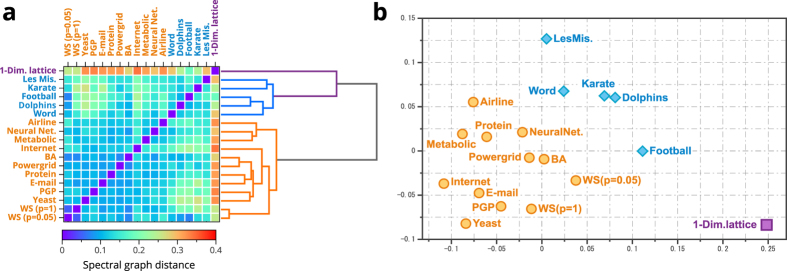
(**a**) Spectral graph distances between real networks and networks obtained from mathematical models. The figure shows the distance matrix, with colours representing the spectral graph distances. The tree shown in Fig. 2a is the hierarchical clustering tree calculated by the classical hierarchical clustering method on the basis of the distance matrix. (**b**) Two-dimensional visualization of networks obtained by multidimensional scaling (MDS)^18^. See Methods and also Supplementary Information for further details.

Next, we apply our method to real networks. Figure 2a shows the distance matrix in which the real networks as well as mathematical models are listed on the vertical and horizontal axes, and colours show their inter-network distances. In Fig. 2b, each network is arranged in the two-dimensional Euclidean space such that their spectral graph distances are preserved as much as possible using multidimensional scaling (see for example ref. 18 and Supplementary Information). The hierarchical tree described on the right-hand side of Fig. 2a is obtained by the classical hierarchical clustering method (see also Methods).

As Fig. 2a,b shows, the real networks are mainly classified on the basis of their spectral graph distances into two groups: one consists of biological and technological networks, and the other consists mainly of social networks, including the dolphin social network, the network of words and the network of football games. These results agree with the previous research^19^, which discussed why the social networks differ from other types of real networks. On the other hand, the one-dimensional lattice is relatively far from other networks because its structure is regular and different from those of many real biological, technological and social networks.

### Analyzing temporal properties in face-to-face human interactions

We next apply our method to temporal networks. One example is a contact network in which face-to-face interactions between human individuals are recorded using radio frequency identifiers^20^,^21^. The temporal network is described as a set of networks 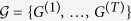, where *G*^(*t*)^ is a set of nodes and links that are observed within a certain period of time from *t* × Δ*t* to (*t* + 1) × Δ*t*. The index, *t*, of *G*^(*t*)^ corresponds to the discrete temporal index. In real contact networks, individuals are likely to have many contacts in the daytime, but few during the night. If we simply focus on temporal changes in the number of links and nodes, these temporal networks seemingly have a one-day cycle (see also Supplementary Information). However, even if the temporal changes in the number of nodes and links are periodic, this does not directly indicate that network structures of the temporal networks also change periodically with time. This periodicity in the structures of the temporal networks is considerably important, especially when we evaluate the dynamical properties of temporal networks, because the structural properties characterised by the Laplacian matrix directly affect the dynamical properties^2^,^14^,^22^.

If the temporal change in the structures of the contact networks is periodic, there exists a non-zero positive constant τ^*^ such that the network structure of *G*^(*t*)^ is equivalent to that of 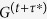 for all values of *t*. We distinguish this periodicity in the structures of the temporal networks from the periodicity simply observed from the temporal changes in the number of nodes and links, and then we call the former *structural periodicity*. The final purpose here is to evaluate the structural periodicity of these real temporal networks.

The spectral graph distance enables us to determine whether the temporal networks have structural periodicity, by evaluating temporal differences between the network, *G*^(*t*)^, and its *m*-nearest neighbours for all values of *t* as follows:


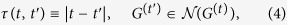


where *t* ≠ *t*′ and 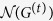 is a set of *m*-nearest neighbours of *G*^(*t*)^ given by the spectral graph distance. If the contact network exhibits structural periodic behaviour, the values of *τ*(*t*, *t*′) should be cτ^*^ (*c* = 1, 2,…), where τ^*^ is its period, but if the contact network exhibits random behaviour, *τ*(*t*, *t*′) takes several values. We simply describe *τ*(*t*, *t*′) as *τ* hereafter.

In the experiments, we apply the proposed method to three types of temporal networks of contacts between individuals observed in a hospital, a high school and a science gallery^20^,^21^,^23^. Each contact network is divided into *T* smaller networks every 60 min, and the number of networks, *T*, in the dataset of the hospital data is 97, that of the high school is 202, and that of the science gallery is 1,929. Evaluating the value of Eq. (4) for the networks *G*^(*t*)^ (*t* = 1, …, *T*), we first obtain the probability distribution of the value of *τ*, *P*(*τ*). Next, we calculate the probability distribution, *P*(*τ*_*r*_), of the temporal difference, *τ*_*r*_, between *G*^(*t*)^ and 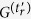, which is randomly selected from 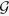. In the real contact networks, due to few contacts in the night time, the contact networks observed in the daytime are likely to be selected, even in the case of random selections, and thereby, the occurrence frequency of *τ* is biased. To avoid such a bias, we calculate the Z score (*P*(*τ*) − 〈*P*(*τ*_*r*_)〉)/*σ*(*τ*_*r*_), where 〈*P*(*τ*_*r*_)〉 is the expected value of *P*(*τ*_*r*_) and *σ*(*τ*_*r*_) is its standard deviation.

**Figure 3:**
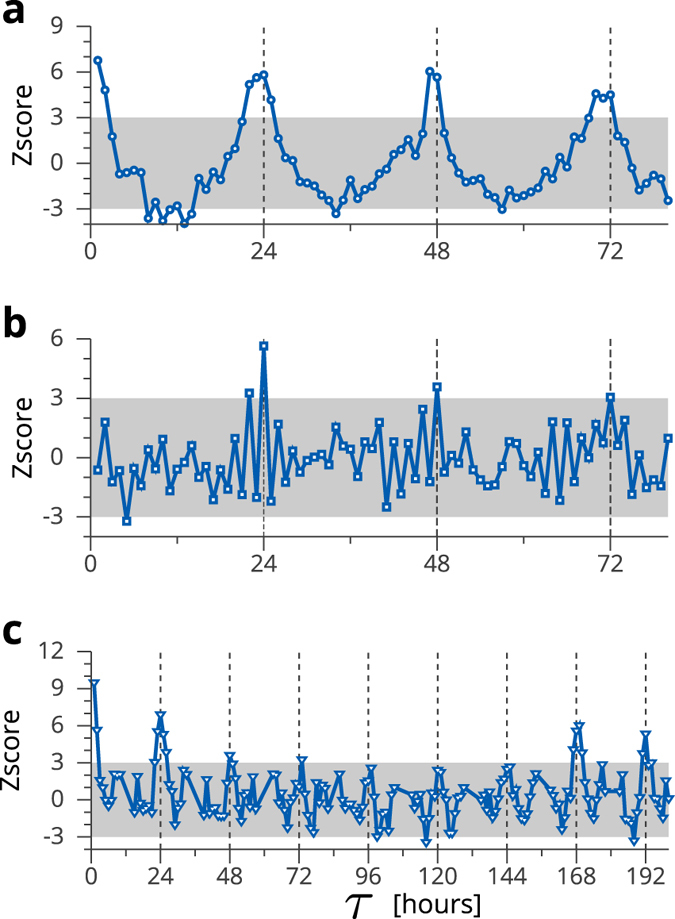
Z score results [*P*(*τ*) − 〈*P*(*τ*_*r*_)〉]/*σ*(*τ*_*r*_) for the hospital (**a**), high school (**b**) and gallery (**c**). The grey shading shows the area where −3 < Z score <3. The number of nearest neighbours, *m*, is set to 20% of the number of networks, *T*. The distances between the networks are calculated using the maximum connected component of *G*^(*t*)^.

From Fig. 3, we find peaks at *τ* = 24, 48 and 72 in the results of the contact networks observed in the hospital and the high school. This result indicates that these contact networks exhibit recurring 24-h patterns. These results agree with intuition; most nodes in the contact network observed in the hospital are staff members including doctors and nurses who work there and patients who are admitted. Many of them participate in the temporal network every day. In the ward, the staff members work according to their fixed schedule and the temporal networks observed in the hospital are likely to exhibit periodicity. In the case of the high school, the students also behave according to a certain fixed schedule. In such cases, the network structure of *G*^(*t*)^ is likely to be similar to that of 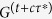, where τ^*^(> 0) is the period of the contact network. The spectral graph distance effectively elucidates the structural periodicity in the contact networks. On the other hand, we also find the peaks at *τ* = 24, 48, 72, 168, and 192 in the results of the science gallery to be against intuition; most nodes in the contact networks observed in the science gallery are visitors and their network structures seem to be randomly varied every day. However, our results reveal that even if the visitors change day by day, the temporal networks exhibit recurring patterns.

In summary, we have proposed the spectral graph distance that can evaluate the distance between complex networks on the basis of their spectra of the Laplacian matrices. The spectral graph distance was successfully applied to the classification of several networks and to the analysis of the contact networks, and has elucidated the temporal property underlying real contact networks, namely that which we call structural periodicity. In particular, revealing temporal properties underlying temporal networks would involve several applications, such as time series analysis of networks^24^,^25^, prediction and prevention of the spread of infectious diseases on temporal networks^26^,^27^,^28^, and elucidation of the relationship between functions and network structures in many real systems^29^,^30^. The spectral graph distance could be quite an important tool for answering these open questions, and carrying out more in-depth discussion on the proper distance between complex networks would give us a novel perspective on the dynamical properties of temporal networks. In addition to these applications, the notion of the spectral distance between networks is important and useful when we assess the properties of networks, for example, quantum networks^31^,^32^. The application of our distance to the quantum networks is one of the most important future works.

## Methods

### Distance between distributions

In Eq. (3), we need to calculate the distances between distributions 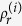 and 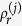, which are the distributions of elements in the *r*th eigenvectors of the Laplacian matrices corresponding to the *i*th and *j*th networks, *G*^(*i*)^ and *G*^(*j*)^. Let 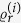 be a cumulative distribution function of the elements in the *r*th eigenvector, 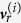, of the Laplacian matrix of the *i*th network. The cumulative distribution function is calculated by 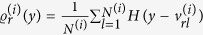, where *H*(*y*) is the step function in which *H*(*y*) is unity if *y* ≥ 0, and zero otherwise. Although there are several choices for the distance between distributions, we use the following:


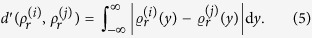


To numerically estimate the distribution function 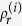, one needs to determine a suitable bin size, but this selection is complicated because of a wide variety of distributions of elements in the eigenvectors obtained from complex networks. However, the cumulative distribution function, 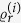, is directly calculated from the elements of the eigenvector 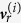 without any parameter. In our method, the elements in the eigenvector are normalised such that their minimum and maximum values are zero and unity. Transforming each eigenvector into the distribution function of its elements enables us to compare two eigenvectors of different sizes.

### Hierarchical clustering

We used a classical hierarchical clustering to classify several complex networks. We first calculated the spectral graph distances between the networks. In the initial step, the individual networks are considered as clusters, that is, each cluster includes only one network. Next, using the obtained spectral graph distances, we merged the two closest clusters until only one cluster remained on the basis of the classical Ward’s method^33^. This hierarchical clustering is visualised by using a tree-like diagram.

We calculated the spectral graph distance between real networks and then applied the classical hierarchical clustering. Here, the real networks are the adjacency network of adjectives and nouns in the *David Copperfield* (Word)^16^, a social network of 62 dolphins (Dolphin)^34^, human relations between 34 members of the Zachary karate club (Karate)^35^, a co-appearance network between 77 characters in the famous novel *Les Miserables* (LesMis)^36^, the neural network of C. elegans (NeuralNet)^17^, the power grid (Powergrid)^17^, the C. elegans metabolic network (Metabolic)^37^, the Internet (Internet)^38^, the e-mail network (E-mail)^39^, the network of users of the Pretty-Good-Privacy algorithm (PGP)^40^, the protein interaction network of yeast (Yeast)^41^, and the airline network (Airline)^42^,^43^. We also used the networks generated from the WS model^17^ and the Barabási–Albert (BA) model^44^. In the WS model, the networks are generated with the rewiring probabilities *p* = 0 (1-Dim. Lattice), *p* = 0.05 (WS(*p* = 0.05)) and *p* = 1 (WS(*p* = 1)), and the initial one-dimensional lattice has *N* = 5,000 nodes, with the degree of each node being 10. In the BA model, a single new node with 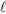 links is repeatedly added to the initial complete graph consisting of 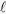 nodes. The network grows until the number of nodes reaches 5,000, and 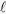 is set to three (BA). The texts in parentheses in the above list of real networks correspond to the labels of real networks in Fig. 2.

## Additional Information

**How to cite this article**: Shimada, Y. *et al*. Graph distance for complex networks. *Sci. Rep*. **6**, 34944; doi: 10.1038/srep34944 (2016).

## Supplementary Material

Supplementary Information
