# Supplementary material for: Graph distance for complex networks
Source: Sci Rep. 2016 Oct 11;6:34944. doi: 10.1038/srep34944 (PMC5057156; doi:10.1038/srep34944)
Supplement: Supplementary Information [file srep34944-s1.pdf]

Supplementary Information: "Graph distance for complex networks"

Yutaka Shimada[1],* Yoshito Hirata[2], Tohru Ikeguchi[1], and Kazuyuki Aihara[2]

[1]*Faculty of Engineering, Tokyo University of Science,*

1-3 Kagurazaka, Shinjuku-ku, Tokyo 162-8601, Japan and

[2]*Institute of Industrial Science, the University of Tokyo,*

4-6-1 Komaba, Meguro-ku, Tokyo 153-8505, Japan

(Dated: March 13, 2016)

Contents

I. Kruglov distance 2

II. Properties of spectral graph distance for complex networks 2

III. Technical details for calculating spectral graph distances 4

IV. Visualisation of distances between networks by multidimensional scaling 5

V. Properties of studied temporal networks 6

VI. An example of how to extract period from distances 10

References 11

*Electronic address: `yshimada@rs.tus.ac.jp`

I. KRUGLOV DISTANCE

To calculate the distance between distributions of eigenvector components, we use the Kruglov distance [1]. Let $\mathbb{P}$ be the set of all distributions. The Kruglov distance is defined by

$$d'(\rho^{(i)}, \rho^{(j)}) = \int_{-\infty}^{\infty} f[\varrho^{(i)}(y) - \varrho^{(j)}(y)]\, \mathrm{d}y, \tag{S1}$$

where f is an even and strictly increasing function on $[0, \infty)$, $f(0) = 0$, and $\varrho^{(i)}$ is a cumulative distribution function of the ith probability distribution $\rho^{(i)} \in \mathbb{P}$. In this paper, we use the absolute value function, $f(x) = |x|$, which is clearly a non-negative, even, strictly increasing function on $[0, \infty)$, and $f(0) = 0$. In this case, Eq. (S1) satisfies the following properties for $\forall \rho^{(i)}, \rho^{(j)}, \rho^{(k)} \in \mathbb{P}$:

(D0) $d'(\rho^{(i)}, \rho^{(j)}) \geq 0$,

(D1) $d'(\rho^{(i)}, \rho^{(j)}) = 0 \;\Leftrightarrow\; \rho^{(i)} = \rho^{(j)}$,

(D2) $d'(\rho^{(i)}, \rho^{(j)}) = d'(\rho^{(j)}, \rho^{(i)})$,

(D3) $d'(\rho^{(i)}, \rho^{(j)}) \leq d'(\rho^{(i)}, \rho^{(k)}) + d'(\rho^{(k)}, \rho^{(j)})$.

II. PROPERTIES OF SPECTRAL GRAPH DISTANCE FOR COMPLEX NETWORKS

As indicated by Eq. (3) and the Methods section in the main text, to evaluate the distance between two given networks $G^{(i)}$ and $G^{(j)}$, we compare the eigenvectors obtained from their Laplacian matrices $L^{(i)}$ and $L^{(j)}$. Let $\mathbb{G}$ be the set of all undirected and unweighted connected graphs. The proposed spectral graph distance satisfies the following conditions for $\forall G^{(i)}, G^{(j)} \in \mathbb{G}$:

(D'0) $d(G^{(i)}, G^{(j)}) \geq 0$,

(D'1) $i = j \Rightarrow d(G^{(i)}, G^{(j)}) = 0$,

(D'2) $d(G^{(i)}, G^{(j)}) = d(G^{(j)}, G^{(i)})$.

These properties are confirmed as follows:

(D'0) For $\forall \rho^{(i)}, \rho^{(j)} \in \mathbb{P}$, $d'(\rho^{(i)}, \rho^{(j)}) \geq 0$, and thus, from the definition of the spectral graph distance (Eq. (3) in the main text), $d(G^{(i)}, G^{(j)}) \geq 0$.

(D'1) When $i = j$, $\rho_r^{(i)} = \rho_r^{(j)}$ for all r. Thus, $i = j \Rightarrow d(G^{(i)}, G^{(j)}) = 0$. In particular, (D'1) implies that there exist two non-identical networks such that $d(G^{(i)}, G^{(j)}) = 0$ $(i \neq j)$.

(D'2) From (D2) in section I and the definition of the spectral graph distance (Eq. (3) in the main text), clearly $d(G^{(i)}, G^{(j)}) = d(G^{(j)}, G^{(i)})$.

We next show that our distance satisfies the triangle inequality when M_{ij} in Eq. (3) in the main text takes a fixed constant value for all network pairs. Let $\mathbb{G}'$ be a set of graphs $\mathbb{G}' \in \mathbb{G}$ such that for $\forall G^{(i)}, G^{(j)} \in \mathbb{G}'$, $d(G^{(i)}, G^{(j)}) = 0 \Leftrightarrow G^{(i)} = G^{(j)}$ and $M_{ij} = M^*$ for all pairs of $G^{(i)}$ and $G^{(j)}$, where M^* is a fixed constant value that satisfies $M^* \leq \min_i(N^{(i)})$. Under this condition, the spectral graph distance satisfies the following triangle inequality:

(D'3) $\forall G^{(i)}, G^{(j)}, G^{(k)} \in \mathbb{G}',\ d(G^{(i)}, G^{(j)}) \leq d(G^{(i)}, G^{(k)}) + d(G^{(k)}, G^{(j)})$.

This property is confirmed using the fact that $d'(\rho_r^{(i)}, \rho_r^{(j)}) - d'(\rho_r^{(k)}, \rho_r^{(j)}) \leq d'(\rho_r^{(i)}, \rho_r^{(k)})$ from (D3) in section I:

$$\begin{aligned}
d(G^{(i)}, G^{(j)}) &= \frac{1}{M^*}\sum_{r=2}^{M^*} d'(\rho_r^{(i)}, \rho_r^{(j)}), \\
&= \frac{1}{M^*}\left\{\sum_{r=2}^{M^*} d'(\rho_r^{(i)}, \rho_r^{(j)}) - \sum_{r=2}^{M^*} d'(\rho_r^{(k)}, \rho_r^{(j)}) + \sum_{r=2}^{M^*} d'(\rho_r^{(k)}, \rho_r^{(j)})\right\}, \\
&= \frac{1}{M^*}\left\{\sum_{r=2}^{M^*} \left[d'(\rho_r^{(i)}, \rho_r^{(j)}) - d'(\rho_r^{(k)}, \rho_r^{(j)})\right] + \sum_{r=2}^{M^*} d'(\rho_r^{(k)}, \rho_r^{(j)})\right\}, \\
&\leq \frac{1}{M^*}\left\{\sum_{r=2}^{M^*} d'(\rho_r^{(i)}, \rho_r^{(k)}) + M^* d(G^{(j)}, G^{(k)})\right\}, \\
&= \frac{1}{M^*}\left\{M^* d(G^{(i)}, G^{(k)}) + M^* d(G^{(j)}, G^{(k)})\right\}, \\
&= d(G^{(i)}, G^{(k)}) + d(G^{(j)}, G^{(k)}).
\end{aligned}$$

From these results, when M^* takes a fixed constant value, the spectral graph distance on $\mathbb{G}'$ satisfies (D'0)–(D'3). In particular, the triangle inequality is more important than the properties (D'0)–(D'2) from the viewpoint of the application. For example, many clustering methods are designed on Euclidean space, and the triangle inequality affects their performance and convergence of their algorithms.

According to our method, the distances between networks are calculated using the cumulative distributions of elements in the eigenvectors corresponding to their Laplacian matrices. In this case, the indices of the nodes do not affect the distance — that is, even if the sets of nodes and links included in the two networks, $G^{(i)}$ and $G^{(j)}$, differ from each other, $d(G^{(i)}, G^{(j)})$ can be zero when these networks have the exact same network structure. In addition, the two Laplacian matrices

$L^{(i)}$ and $L^{(j)}$ can share the distributions of their eigenvectors. For these reasons, the spectral graph distance is not a strict distance, but a quasi-distance.

III. TECHNICAL DETAILS FOR CALCULATING SPECTRAL GRAPH DISTANCES

Even though the triangle inequality is satisfied in the case where $M_{ij} = M^* = \min_i N^{(i)}$, M^* might become too small to precisely evaluate distances between networks when the set of networks $\mathbb{G}'$ includes one whose size is too small relative to the other networks contained in $\mathbb{G}'$. In this sense, it is more effective to employ the strategy that $M_{ij} = \min(N^{(i)}, N^{(j)})$, where the value of M_{ij} is adaptively determined on the basis of the sizes of two networks $G^{(i)}$ and $G^{(j)}$. However, in this strategy, there is no guarantee that the proposed spectral graph distance satisfies the triangle inequality. If this inequality is not satisfied, the clustering method might lead to an erroneous result. To avoid such a situation, we need to check whether the distances, $d(G^{(i)}, G^{(j)})$, obtained from a given set of networks, $\mathbb{G}'$, satisfy the triangle inequality. In the following, we show an approach to accomplishing this aim.

Let D be an $n \times n$ distance matrix whose (i,j)th element is the spectral graph distance $d(G^{(i)}, G^{(j)})$ between networks $G^{(i)}$ and $G^{(j)}$ $(i, j = 1, \cdots, n)$. Because the spectral graph distance always satisfies the conditions (D'0)–(D'2), we can use the well-known result that *D is Euclidean $\Leftrightarrow$ PDP is negative semi-definite* (see for example [2]), where the matrix P is defined by

$$P = I_n - \frac{1}{n}\mathbf{1}_n\mathbf{1}_n^\intercal, \tag{S2}$$

I_n is the $n \times n$ identity matrix, and $\mathbf{1}_n = (1, \cdots, 1)^\intercal$ is a vector with n ones. In other words, D is Euclidean when the minimum eigenvalue of PDP is not positive. In addition, even if PDP is not negative semi-definite, adding a fixed constant κ to the off-diagonal elements of D, we can obtain a transformed Euclidean distance matrix D_κ as follows [3]:

$$D_\kappa = D + \kappa(\mathbf{1}_n\mathbf{1}_n^\intercal - I_n). \tag{S3}$$

The value of κ is selected such that the eigenvalues of $PD_\kappa P$ are not positive. One simple approach is to use the maximum eigenvalue of PDP as the value of κ, because the ith eigenvalue μ_i of PDP shifts from μ_i to $\mu_i - \kappa$ by adding κ to the off-diagonal elements of PDP. Indeed,

$$PD_\kappa P \;=\; P\left[D + \kappa(\mathbf{1}_n\mathbf{1}_n^\intercal - I_n)\right]P = PPDPP - \kappa PP = P(PDP - \kappa I_n)P = PU(\mathcal{M} - \kappa I_n)U^\intercal P, \tag{S4}$$

where $\mathcal{M} = \mathrm{diag}(\mu_1, \ldots, \mu_n)$, $U = (\boldsymbol{u}_1, \ldots, \boldsymbol{u}_n)$, and $\boldsymbol{u}_i = (u_{1i}, \ldots, u_{ni})^\intercal$ is the eigenvector of PDP corresponding to μ_i. The eigenvectors of $PD_\kappa P$ are directly obtained from PU. In Eq. (S4), we have used $P\mathbf{1}_n = \mathbf{0}_n$ and $PP = P$, where $\mathbf{0}_n = (0, \ldots, 0)^\intercal$ is a vector with n zeros.

Using this method, we can confirm whether the given networks can be embedded into the Euclidean space such that their distances are satisfied. If the networks can be embedded in Euclidean space, the distance matrix obtained from the spectral graph distances between networks satisfies the triangle inequality. In Fig. 2**a** in the main text, the distance matrix, D, is obtained from 14 real networks and four networks generated from mathematical models. Applying Eq. (S3) to D, we find that the maximum eigenvalue of PDP is 0.0547. Using $\kappa = 0.0547$, the distance matrix D is transformed by Eq. (S3), and the resultant hierarchical tree and the arrangements on two-dimensional Euclidean space are shown in Fig. 2 in the main text.

As a final remark in this section, we note that there are some choices of eigenvectors of the Laplacian matrix. The Laplacian matrix, L, is transformed into its eigenvalues and their eigenvectors,

$$L = V\Lambda V^\intercal, \tag{S5}$$

where $\Lambda = \mathrm{diag}(\lambda_1, \ldots, \lambda_N)$ and $V = (\boldsymbol{v}_1, \ldots, \boldsymbol{v}_N)$. In general, there are 2^N matrices $V = (\pm\boldsymbol{v}_1, \pm\boldsymbol{v}_2, \ldots, \pm\boldsymbol{v}_N)$ such that $L = V\Lambda V^\intercal$. This property of the eigenvectors affects the distribution, ρ_r, of the elements in the rth eigenvector of L. For this reason, the distance $d(G^{(i)}, G^{(j)})$ might become incorrect without careful selection of the signs of the elements in the eigenvectors. To avoid this undesirable effect, the spectral graph distance defined by Eq. (3) in the main text is rewritten by

$$d(G^{(i)}, G^{(j)}) = \frac{1}{M_{ij} - 1} \sum_{r=2}^{M_{ij}} \left\{ \min_{s,l \in \{-1,+1\}} d' \left[\rho(s\boldsymbol{v}_r^{(i)}), \rho(l\boldsymbol{v}_r^{(j)}) \right] \right\}, \tag{S6}$$

In Eq. (S6), the distribution of the elements in the rth eigenvector of $L^{(i)}$ is redefined by $\rho(\boldsymbol{v}_r^{(i)})$ to emphasize the sign of eigenvectors.

IV. VISUALISATION OF DISTANCES BETWEEN NETWORKS BY MULTIDIMENSIONAL SCALING

To visualise the spectral graph distances among networks, we employ multidimensional scaling (MDS) [2]. In Fig. 2**b** in the main text, the networks are arranged in two-dimensional Euclidean space using MDS, such that the spectral graph distance, $d(G^{(i)}, G^{(j)})$, between the networks $G^{(i)}$

and $G^{(j)}$ is approximated by the Euclidean distance $\|\boldsymbol{x}_i - \boldsymbol{x}_j\|$ as closely as possible, where $\boldsymbol{x}_i$ is the two-dimensional vector representing the arrangement of $G^{(i)}$ on the Euclidean space. The coordinate values $\boldsymbol{x}_i$ $(i = 1, \cdots, n)$ are determined by minimising the following function S, which is the sum of the square errors between $d(G^{(i)}, G^{(j)})$ and $\|\boldsymbol{x}_i - \boldsymbol{x}_j\|$ for all $d(G^{(i)}, G^{(j)})$:

$$S(\boldsymbol{x}_1, \ldots, \boldsymbol{x}_n) := \left\{ \alpha \sum_{i<j} \left(d(G^{(i)}, G^{(j)}) - \|\boldsymbol{x}_i - \boldsymbol{x}_j\| \right)^2 \right\}^{\frac{1}{2}}, \tag{S7}$$

where $\alpha = \left[\sum_{i<j} d(G^{(i)}, G^{(j)})^2 \right]^{-1}$. The function $S(\boldsymbol{x}_1, \ldots, \boldsymbol{x}_n)$ is minimised by directly applying the gradient descent to Eq. (S7) [2].

Figure S1 shows the resultant scatter plot of $d(G^{(i)}, G^{(j)})$ and $\|\boldsymbol{x}_i - \boldsymbol{x}_j\|$ corresponding to Fig. **2b** in the main text. Figure S1 shows that the spectral graph distance is well-approximated by the Euclidean distance, and that the correlation coefficient between $d(G^{(i)}, G^{(j)})$ and $\|\boldsymbol{x}_i - \boldsymbol{x}_j\|$ is 0.93

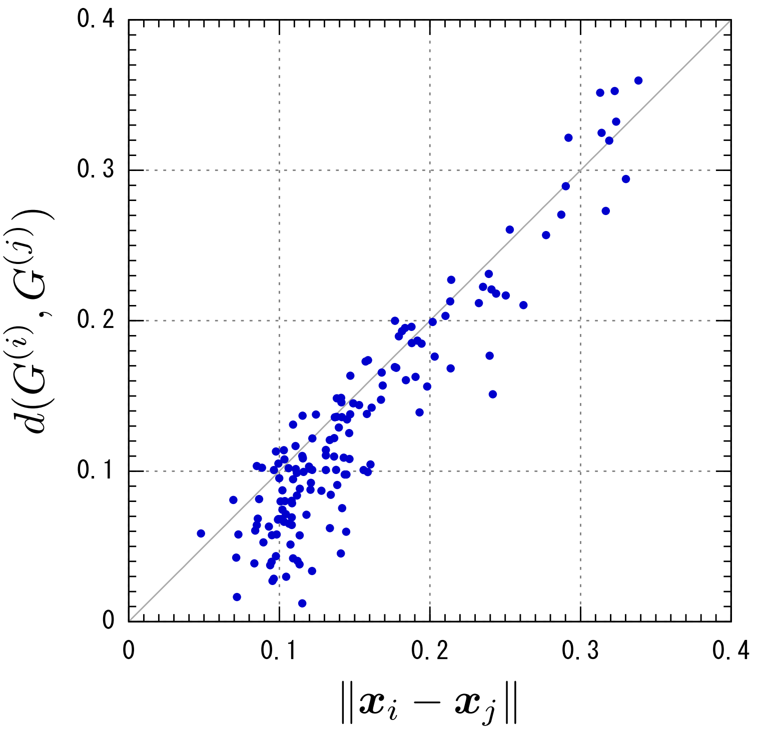

FIG. S1: Scatter plot of $d(G^{(i)}, G^{(j)})$ and $\|\boldsymbol{x}_i - \boldsymbol{x}_j\|$. The correlation coefficient between $d(G^{(i)}, G^{(j)})$ and $\|\boldsymbol{x}_i - \boldsymbol{x}_j\|$ is 0.93. The grey diagonal line is a guide for eyes.

V. PROPERTIES OF STUDIED TEMPORAL NETWORKS

In the main text, we applied the proposed method to three types of temporal networks of contacts between individuals, which were observed in a hospital, a high school and a science gallery,

where workers, students and visitors were in contact with one another [4–6]. These datasets were collected by SocioPatterns collaboration [7] and recorded using radio frequency identifiers. In these datasets, the contacts between two individuals were recorded at a 20-s interval when they came within a certain distance of each other (1–1.5 m). The dataset observed in the hospital was collected over five days [6], that in the high school was collected over seven school days [5] and that in the science gallery was collected over 69 days [4].

In our numerical simulations, the contact data between individuals were divided into T small segments every Δt min, and then T temporal networks were constructed by accumulating the contacts included in each temporal interval $[(t-1)\times\Delta t,\ t\times\Delta t]$ $(t = 1, \cdots, T)$. Then the dataset of contacts between individuals was described as a set of networks, $\mathcal{G} = \{G^{(1)}, \ldots, G^{(T)}\}$, where $G^{(t)}$ is a set of nodes and links that are observed within a certain period from $(t-1)\times\Delta t$ to $t\times\Delta t$. The index t of $G^{(t)}$ corresponds to the discrete temporal index.

When $\Delta t = 60$ min, the number of networks, T, in the contact data of the hospital was 95, that for the high school was 202 and that for the science gallery was 1,928. In the real contact networks, the individuals are likely to have many contacts in the daytime, but few during the night. In fact, the number of links in the daytime increases, but the night drastically decreases in Figs. S2 and S3. If we simply focus on temporal changes in the number of links and nodes, these temporal networks seemingly have a one-day cycle. Table I shows the basic properties of these contact networks. In the numerical simulations, we simply focus on the maximum connected component in each network and then calculate the distances between them. The numbers of nodes in the original temporal networks and in the maximum connected component are shown in Figs. S2 and S3, respectively.

TABLE I: Basic properties of the temporal networks.

Data set	The number of individuals	The number of contacts
Hospital	75	32424
High school	180	45047
Science gallery	10972	415912

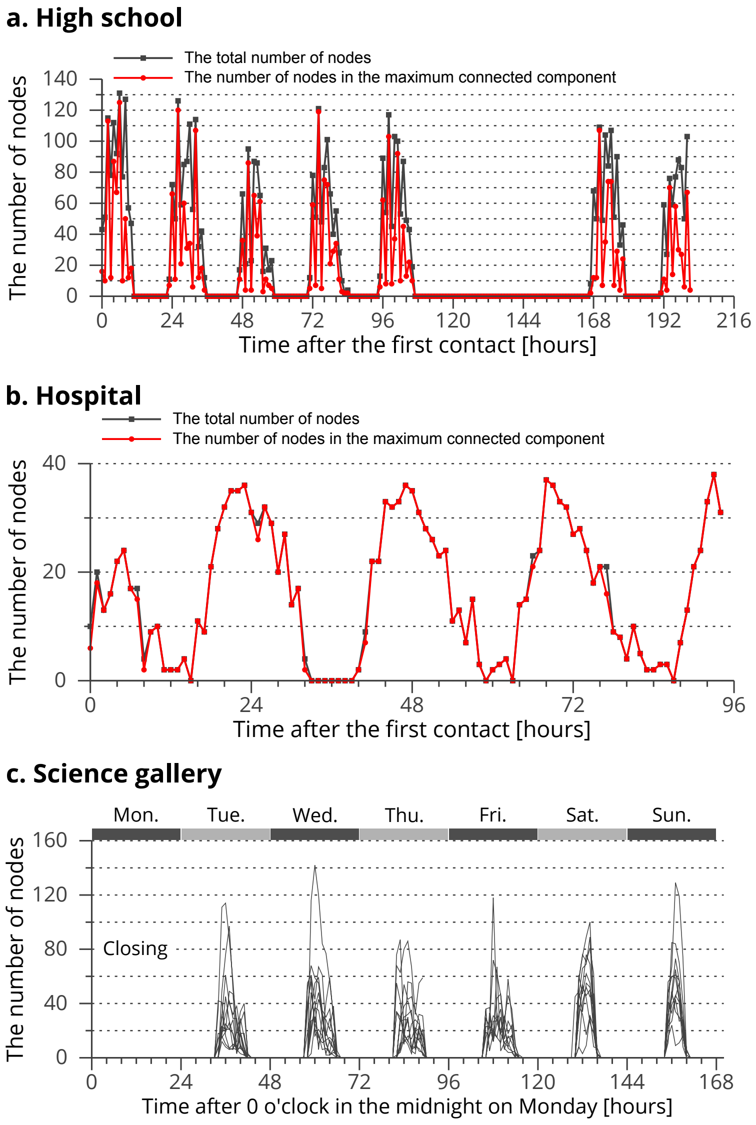

FIG. S2: Temporal changes in the number of individuals who appear in the tth network $G^{(t)}$. The dataset for the science gallery was observed over about 10 weeks, and the weekly temporal changes in the number of visitors are depicted in **c**. The grey lines show the number of nodes included in each network $G^{(t)}$ at time t, and the red lines show the number of nodes included in its maximum connected component.

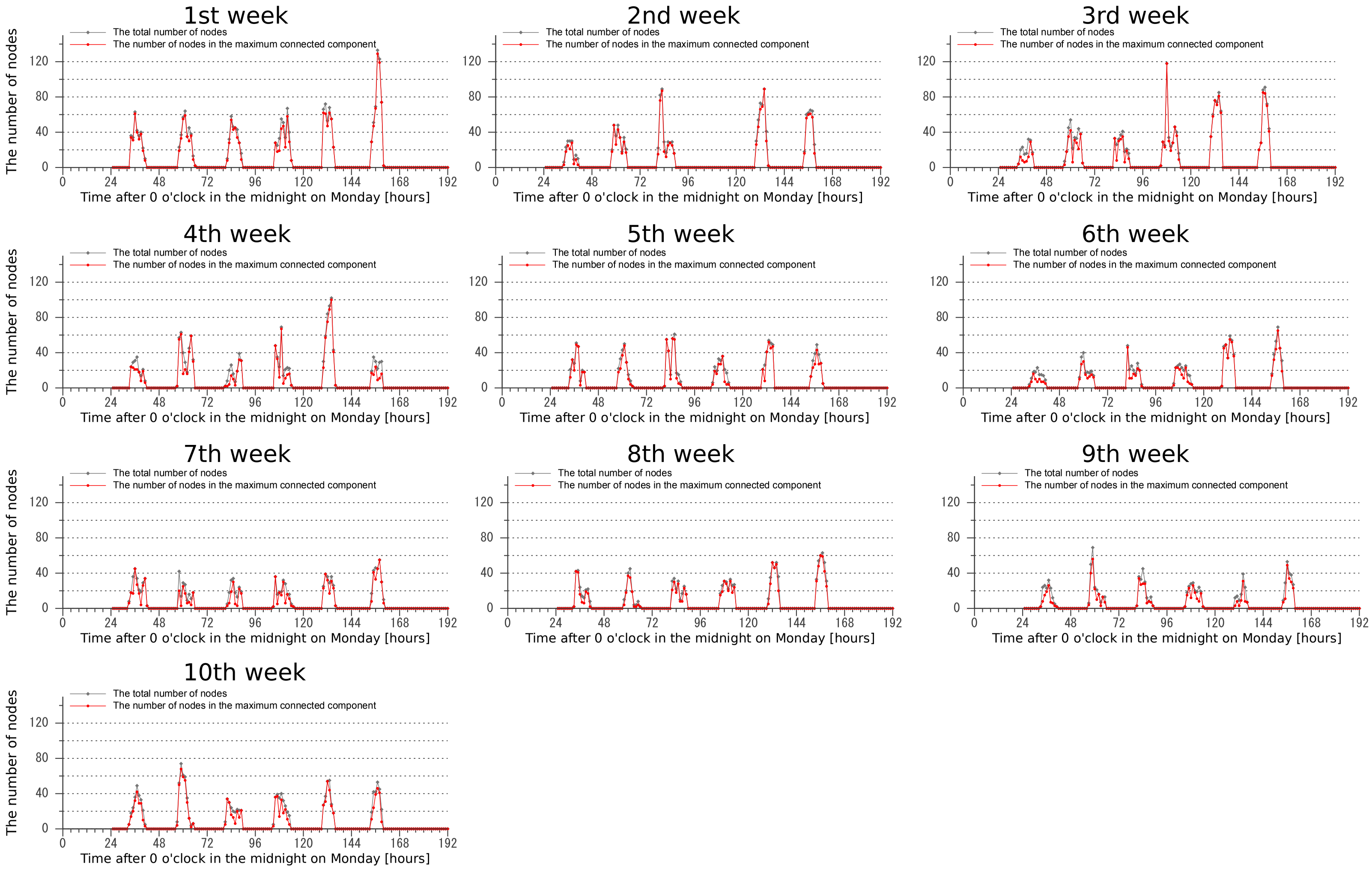

FIG. S3: Temporal changes in the number of visitors for each week. The grey lines show the number of nodes included in each network, $G^{(t)}$, at time t, and the red lines show the number of nodes included in the maximum connected component.

VI. AN EXAMPLE OF HOW TO EXTRACT PERIOD FROM DISTANCES

If an observed multidimensional time-series, $\boldsymbol{x}(t)$, is periodic, there exists a non-zero positive constant, τ^*, such that $\boldsymbol{x}(t)$ is equivalent to $\boldsymbol{x}(t+\tau^*)$ for all values of t. The simplest way to estimate its period, τ^*, using inter-point distances alone is to evaluate temporal differences between $\boldsymbol{x}(t)$ and its m-nearest neighbours as follows:

$$\tau(t,t') \equiv |t-t'|, \;\; \boldsymbol{x}(t') \in \mathcal{N}(\boldsymbol{x}(t)), \tag{S8}$$

where $t \neq t'$ and $\mathcal{N}(\boldsymbol{x}(t))$ is the set of m-nearest neighbours of $\boldsymbol{x}(t)$. If the time series exhibits periodic behaviour, the values of $\tau(t,t')$ should be close to $c\tau^*$ $(c = 1, 2, \ldots)$, where τ^* is its period, but if the time series exhibits random behaviour, $\tau(t,t')$ takes several values. We simply describe $\tau(t,t')$ as τ hereafter.

To check the validity of the above method, we conducted some numerical experiments using the Rössler system described by the following dynamics

$$\begin{aligned} \dot{x} &= -(y+z), \\ \dot{y} &= x + ay, \\ \dot{z} &= b + z(x-c). \end{aligned} \tag{S9}$$

The values of the parameters are set to $a = b = 0.2,\ c = 2.5$, and the three-dimensional periodic orbit is generated as shown in Fig. S4**a**. In addition, in Fig. S4**b**, we add noises $\gamma\xi_x,\ \gamma\xi_y,\ \gamma\xi_z$ to $x(t),\ y(t)$ and $z(t)$, where $\xi_x,\ \xi_y$ and ξ_z represent the observable noises obeying the uniform distribution in $[0,1]$ and γ is the strength of noise that is set to two. From Fig. S4**a** and **b**, the period τ^* is 191. We also depict the random time series $[\xi_x(t),\ \xi_y(t),\ \xi_z(t)]$ on the three-dimensional space in Fig. S4**c**.

Let $\boldsymbol{x}(t) = [x(t),\ y(t),\ z(t)]$ be a point on the periodic orbit at time t. In the numerical simulations, we first calculate the inter-point distances between points on the three-dimensional periodic orbit (Fig. S4**a**), those on the three-dimensional periodic orbit with the noises, namely $\boldsymbol{x}(t) + \boldsymbol{\xi}(t) = [x(t) + \xi_x(t),\ y(t) + \xi_y(t),\ z(t) + \xi_z(t)]$ (Fig. S4**b**), and those on the random time series $\boldsymbol{\xi}(t) = [\xi_x(t),\ \xi_y(t),\ \xi_z(t)]$ (Fig. S4**c**). We then calculate the distribution, $P(\tau)$, of the temporal difference, $\tau = |t - t_s|$, between $\boldsymbol{x}(t)$ and its m-nearest neighbours, $\boldsymbol{x}(t_s)$ $(s = 1, \ldots, m)$. In the same manner, we also calculate $P(\tau_r)$ which is the distribution of the temporal difference $\tau_r = |t - t'_s|$ between $\boldsymbol{x}(t)$ and m randomly chosen points $\boldsymbol{x}(t'_s)$ $(s = 1, \ldots, m)$ for all points $\boldsymbol{x}(t)$. We repeatedly calculate $P(\tau_r)$ 1,000 times and then obtain the expected value $\langle P(\tau_r)\rangle$ and its standard deviation $\sigma(\tau_r)$. The Z score is finally calculated by $[P(\tau) - \langle P(\tau_r)\rangle]/\sigma(\tau_r)$ as a qualitative measure

of statistical significance. Figure S4 **g**, **h**, and **i** shows the resultant Z scores. When the value of τ is close to the period of $\boldsymbol{x}(t)$, the Z score shows clear peaks in both cases with and without noise (Fig. S4**g** and **h**). On the other hand, we cannot find any significant peaks in the case of the random time series (Fig. S4 **i**).

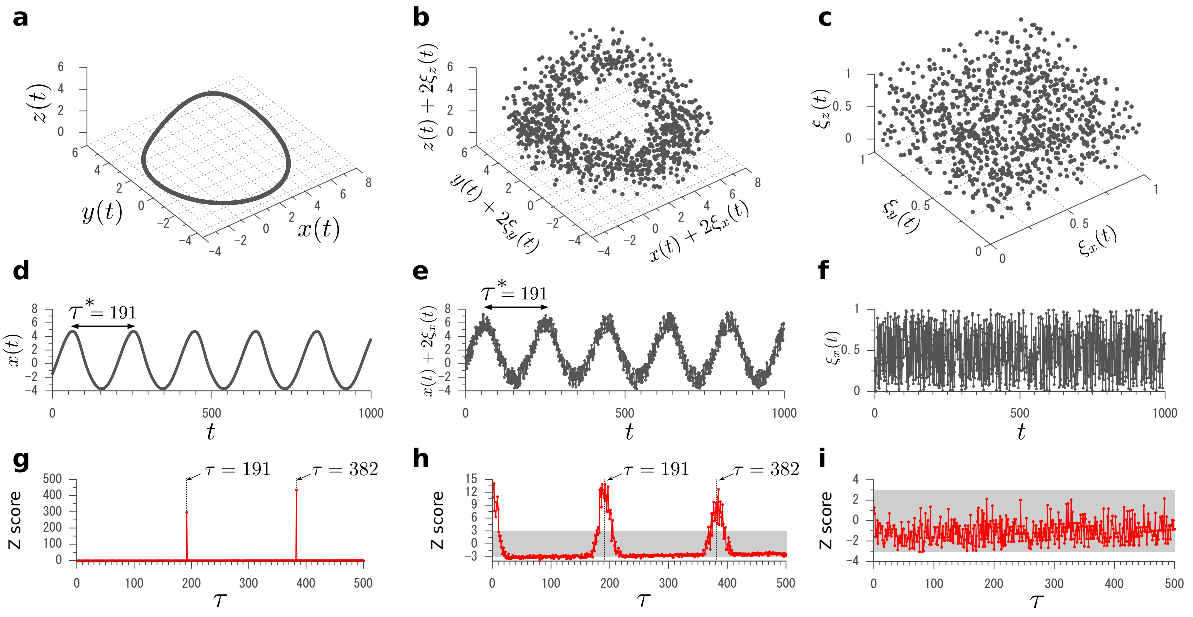

FIG. S4: **a**, The periodic orbit generated from the Rössler system $\boldsymbol{x}(t)$. **b**, The noisy periodic orbit obtained by adding the observable noises to $\boldsymbol{x}(t)$. **c**, The random time series $(\xi_x(t),\ \xi_y(t),\ \xi_z(t))$, where $\xi_x(t),\ \xi_y(t),\ \xi_z(t)$ obey the uniform distribution in $[0, 1]$. **d**, The periodic time series $x(t)$. **e**, The periodic time series with noise $x(t) + \xi_x(t)$. **f**, The random time series $\xi_x(t)$. **g**, **h**, **i**, The results of the Z score $[P(\tau) - P(\tau_r)]/\sigma(\tau_r)$. The grey shading shows the area where $-3 <$ Z score < 3. The number of nearest neighbours m is set to five.

---

[1] Deza, M. M. & Deza, E. *Encyclopedia of distances* (Springer-Verlag Berlin Heidelberg, 2013), 2 edn.

[2] Cox, T. & Cox, M. *Multidimensional scaling.* (Chapman & Hall/CRC, Boca Raton, 2001).

[3] Hathaway, R. J. & Bezdek, J. C. Nerf c-means: Non-Euclidean relational fuzzy clustering. *Pattern Recogn.* **27**, 429–437 (1994).

[4] Isella, L. *et al.* What's in a crowd? Analysis of face-to-face behavioral networks. *J. Theor. Biol.* **271**, 166–180 (2011).

[5] Fournet, J. & Barrat, A. Contact patterns among high school students. *PLoS ONE* **9**, e107878 (2014).

[6] Vanhems, P. *et al.* Estimating potential infection transmission routes in hospital wards using wearable proximity sensors. *PLoS ONE* **8**, e73970 (2013).

[7] `http://www.sociopatterns.org` (accessed October 10, 2015).
